# Evaluating the real-world usability of BCI control systems with augmented reality: a user study protocol

**DOI:** 10.3389/fnhum.2024.1448584

**Published:** 2024-08-05

**Authors:** Arnau Dillen, Mohsen Omidi, María Alejandra Díaz, Fakhreddine Ghaffari, Bart Roelands, Bram Vanderborght, Olivier Romain, Kevin De Pauw

**Affiliations:** ^1^Human Physiology and Sports Physiotherapy Research Group, Vrije Universiteit Brussel, Brussels, Belgium; ^2^Équipes Traitement de l'Information et Systèmes, UMR 8051, CY Cergy Paris Université, École Nationale Supérieure de l'Électronique et de ses Applications (ENSEA), Centre national de la recherche scientifique (CNRS), Cergy, France; ^3^Brussels Human Robotic Research Center (BruBotics), Vrije Universiteit Brussel, Brussels, Belgium; ^4^imec, Brussels, Belgium

**Keywords:** brain-computer interface, eye tracking, augmented reality, robot control, user evaluation, user experience, human-robot interaction

## Abstract

Brain-computer interfaces (BCI) enable users to control devices through their brain activity. Motor imagery (MI), the neural activity resulting from an individual imagining performing a movement, is a common control paradigm. This study introduces a user-centric evaluation protocol for assessing the performance and user experience of an MI-based BCI control system utilizing augmented reality. Augmented reality is employed to enhance user interaction by displaying environment-aware actions, and guiding users on the necessary imagined movements for specific device commands. One of the major gaps in existing research is the lack of comprehensive evaluation methodologies, particularly in real-world conditions. To address this gap, our protocol combines quantitative and qualitative assessments across three phases. In the initial phase, the BCI prototype's technical robustness is validated. Subsequently, the second phase involves a performance assessment of the control system. The third phase introduces a comparative analysis between the prototype and an alternative approach, incorporating detailed user experience evaluations through questionnaires and comparisons with non-BCI control methods. Participants engage in various tasks, such as object sorting, picking and placing, and playing a board game using the BCI control system. The evaluation procedure is designed for versatility, intending applicability beyond the specific use case presented. Its adaptability enables easy customization to meet the specific user requirements of the investigated BCI control application. This user-centric evaluation protocol offers a comprehensive framework for iterative improvements to the BCI prototype, ensuring technical validation, performance assessment, and user experience evaluation in a systematic and user-focused manner.

## 1 Introduction

Brain-computer interfaces (BCI) allow users to operate a device with their brain activity. This enables intuitive control for applications where classical control modalities such as mouse and keyboard are impractical due to either the environment or the user. For example, BCI can be used to enable individuals who would otherwise not be able to interact with their environment, such as patients suffering from locked-in syndrome, to operate assistive devices (Saeedi et al., 2017; Kuhner et al., 2019). These types of applications aim to improve the autonomy of the user and subsequently improve their quality of life by making them less dependent on caregivers for daily activities that require reaching, grasping, and placing objects in the environment. One such application involves physically assistive robots that assist paralyzed individuals in performing daily tasks such as eating, drinking, and interacting with objects in their environment (Selvaggio et al., 2021). To optimally assist users, BCI control systems should provide a good user experience by minimizing the required mental effort and avoiding frustration with a reliable and user-friendly user interface (UI).

**Figure 1:**
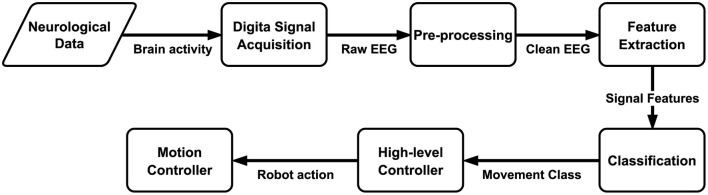
The BCI decoding pipeline for high-level robot control based on the classification of neural activity.

BCI control is achieved by measuring brain activity and decoding the user's intent from the acquired brain signals. Decoding the user's intent from brain signals is typically accomplished by using machine learning (ML) methods that are trained on data of users performing a specific task while their neural activity is measured (Lebedev and Nicolelis, 2017). During real-time decoding, the ML model's output is subsequently translated into a command or action for the specific device or software targeted by the BCI system, such as a physically assistive robot. Figure 1 shows an overview of the typical pipeline for BCI control of robotic devices.

One of the most commonly used recording methods for BCI is the electroencephalogram (EEG), which non-invasively measures the electrical activity at the surface of the scalp. EEG is a favored choice for BCI due to its portability, relative affordability, and high temporal resolution compared to alternative non-invasive brain measures such as functional near-infrared spectroscopy or magnetoencephalography (Gu et al., 2021). There are multiple paradigms of the EEG signal that can be used for BCI control (Abiri et al., 2019). One common paradigm is motor imagery (MI), the mental activity that occurs when a person imagines performing a movement without executing it (Singh et al., 2021). Conventionally, MI is defined as “the mental simulation of an action without the corresponding motor output” (Decety, 1996).

There are three discernible approaches to simulating a movement to generate MI. The first approach involves imagining the sensation of performing the movement, termed kinesthetic MI (Cumming and Ste-Marie, 2001). Alternatively, the movement can be simulated by visualizing it either from one's own perspective (internal visual MI) or by picturing someone else executing the movement (external visual MI) (Cumming and Ste-Marie, 2001). The main advantage of using MI is that it is a spontaneous modality that can be initiated by the user at any time. By associating different actions with specific imagined movements, a BCI control system can be devised. While MI has the advantage of being a spontaneous paradigm, using MI for BCI control applications comes with several challenges (Singh et al., 2021).

One important challenge is that EEG is a noisy signal that is sensitive to movement-induced artifacts (Gorjan et al., 2022). Additionally, EEG is a non-stationary signal with a large inter- and intra-individual variability (Saha and Baumert, 2020). Therefore, to be able to decode EEG reliably, decoding pipelines usually need to be customized to the current user by acquiring sufficient calibration data and identifying the settings of the decoding pipeline, such as which sensor locations to use or which frequencies to filter out of the EEG signal, that are optimal for a specific individual (Dillen et al., 2023). Furthermore, MI consists of consciously generating neural activity that usually occurs unconsciously while performing a movement. Consequently, the users themselves typically also need training to learn to consistently generate the correct signals to enable reliable decoding (Roc et al., 2021). Thus, an approach is necessary for mutual training of the user and the ML models, but this is a lengthy process that necessitates several training sessions (Hehenberger et al., 2021). These limitations restrict the number of movements that can be used for MI-based BCI control systems and raise the barrier of entry for new users, resulting in a suboptimal user experience.

Each step of the BCI pipeline offers multiple opportunities to improve MI-based control. Sophisticated EEG pre-processing methods can be employed to ensure that the signal contains as few artifacts as possible (Saba-Sadiya et al., 2021; Gorjan et al., 2022). Some research attempts to increase the number of commands by using advanced ML methods such as deep learning that can classify multiple imagined movements (Al-Saegh et al., 2021). Others aim to reduce the amount of data that is necessary to train user-specific decoding models, which is especially challenging for data-hungry deep learning models, by using transfer learning (Wan et al., 2021). However, these methods are usually computationally complex, introducing additional processing and increased power consumption which are not practical for real-time applications, especially if the decoding should take place on embedded hardware, which is preferable for real-world applications (Belwafi et al., 2017).

A strategy that shows particular promise without relying on complex and potentially computationally expensive methods is shared control (Philips et al., 2007; Tonin et al., 2010). By using shared control, the high-level control system can be simplified while still retaining the ability to operate in complex environments. A shared control system can propose actions based on the current state of the device that is being controlled and the environment, which can be deduced from sensors such as depth cameras (Xu et al., 2019). The number of actions, and therefore the number of MI classes that need to be decoded, can be restricted in this way. Combining this with systems that can also deduce the user's current object of focus, for example with eye tracking, can even further restrict the number of action choices by only proposing actions related to the object that the user is currently focusing on (Xu et al., 2022). Moreover, this approach can be complemented with advanced methods for pre-processing and decoding to yield a robust and user-friendly BCI control system (Choi et al., 2023).

However, current research is often limited in its evaluation procedures. Most studies are limited to offline validation of the classification accuracy of their decoding method or a technical evaluation that shows that the developed control system works as intended (Rashid et al., 2020). Few studies that propose a new BCI control system evaluate their prototype outside of the lab or assess the user experience of their approach. Those that do are often not representative of real-world conditions (Dillen et al., 2022). Usability, determined by the efficiency, effectiveness, and user experience of the control system, is seldom evaluated (Ortega-Gijon and Mezura-Godoy, 2019). This leads to limited applicability in real-world conditions and a lack of knowledge of the user requirements for such a system. Taking a user-centered approach should be a central part of the evaluation to guarantee that the control system fulfills the user's needs and provides optimal usability (Kübler et al., 2014; Garro and McKinney, 2020).

Moreover, no standard evaluation procedures exist for comparing the performance and user experience of different control system designs, nor for assessing the added value of BCI compared to non-BCI alternatives. For instance, eye tracking can be used to operate an assistive robotic arm (Cio et al., 2019). With this method, users select commands by focusing their gaze on a menu item in a graphical UI or an object in the environment for a specified duration, prompting the assistive robot to take action. However, eye tracking has drawbacks, such as causing eye strain and requiring constant use of glasses, which can potentially cause eye strain and render the control system unusable if the user has an eye infection. Additionally, in tasks requiring the operator to maintain a fixed gaze, such as assembly tasks, eye tracking is at a disadvantage because the user must look away to select an action. Quantitative measures are essential to determine whether BCI offers similar or better usability when compared with eye tracking or if the evaluated prototype shows improvements over previous designs.

The primary research question of this study is whether using BCI enhances the usability of a shared control system that integrates augmented reality (AR) and eye tracking. The main goal of this user evaluation is to measure the control system's performance and user experience, with a secondary aim of identifying potential design improvements to further enhance usability. Due to the lack of comparable studies, no target metrics exist to gauge improvement over the current state-of-the-art in usability. Therefore, we initially use eye tracking to establish a usability benchmark that our BCI control system aims to surpass. Quantitative measures that can be used to compare usability outcomes meaningfully are suggested to ensure standardized results that can be compared with future studies that use this protocol. The presented protocol will be used in upcoming user studies to evaluate a previously developed BCI prototype. This evaluation procedure is intended to help BCI researchers focus on prototype design and gain new insights from these evaluations, ultimately speeding up progress toward practical real-world applications.

## 2 Methods and analysis

### 2.1 Research design

The study consists of three distinct phases with an initial technical validation of the prototype (Phase 1), followed by two user studies with healthy individuals that aim to assess the user experience of our proposed BCI control system (Phase 2 and Phase 3). The user studies are designed according to the principles of empirical evaluation in the field of human-computer interaction (MacKenzie, 2012). New participants are recruited for each phase and the goals of each phase are distinct.

The participants in this study are able-bodied participants who have no prior experience using a BCI control system. Detailed inclusion and exclusion criteria for participants and a priori sample size analyses for each phase are described in Section 2.2. There is no blinding of either participants or research staff, and all participants perform all tests. The order of the tasks is randomized within each session to ensure that the influence of previous experience and fatigue resulting from previous tasks does not affect the overall results. Regular breaks between tasks are included in the procedure to ensure that participants stay motivated and to minimize the effects of fatigue on their task performance.

A detailed schedule with timing estimates for each session in the different phases, based on durations observed in pilot experiments, is provided in Section 2.3.4. Details on the hardware and software used are discussed in Section 2.3.1. Table 1 provides an overview of the different phases, their goal, the evaluation environment, the sessions that each participant completes, and their main outcome.

**Table 1:** Description of the different phases of the study.

**Phase**	**Phase 1: validation**	**Phase 2: pilot user study**	**Phase 3: full user study**
Goal	Validate the first prototype and ensure that it works as intended	Test evaluation protocol and iterate design of control system	Evaluate the user experience of the control system
Environment	Fully simulated with AR	Initially simulated with AR and using the real robot for the last session	Real interactions with the physical robot and objects
Sample size	3	5	20
Sessions	• Familiarization and calibration (2–3 h) • First test with simple pick-and-place task (2 h)	• Familiarization, calibration and pre-study questionnaires (2–3 h) • Activities of daily living tasks followed by system customization (2 h) • Activities of daily living tasks followed by user experience questionnaire (2 h)	• Familiarization, calibration, and pre-study questionnaires (2–3 h) • Activities of daily living tasks and user training (2 h) • Activities of daily living tasks followed by user experience questionnaire (2 h 30 min - 3 h)
Main outcome	Working BCI control prototype	Final evaluation procedure and improved control system	Final proof-of-concept user experience assessment

Phase 1 is the validation phase as its main goal is to validate that the current BCI control system prototype works as intended. This phase is restricted in the number of participants and only part of the procedure is performed. User experience and system performance outcomes are subsequently used to improve the control system for the next phase. For Phase 2, the full procedure is executed to assess the initial user experience and identify potential further improvements in both the control system and evaluation environments. Finally, Phase 3 consists of a full-scale user study with a real robot being used in a real environment. In this phase, more tasks are performed and the BCI control system is compared with an eye-tracking-based control system.

### 2.2 Participants

The participants of this study are all able-bodied individuals who are recruited for the specific purpose of this study. They have no prior experience with an MI-based BCI control system as we want to assess the experience of first-time users. Recruitment is achieved using posters, flyers, and social media posts that call for participants. The recruitment period for the first phase started on the 4th of December 2023 and ends whenever the necessary number of participants is attained. If participants drop out, a replacement will be recruited as necessary. The inclusion and exclusion criteria for prospective participants are shown in Table 2.

**Table 2:** Participant inclusion and exclusion criteria.

**Inclusion**	**Exclusion**
	Injury before the start of the experiment
Able-bodied participants	Illness within a week before the start of the experiment
Aged between 18 and 60 years	Serious mental and physical disabilities
All sexes will be included	The participant does not understand Dutch, French, or English
	Previous experience with BCI control systems

New participants are recruited for each phase to evaluate the experience of users who are not yet familiar with BCI and enhance the control system between phases. Each phase uses a more comprehensive procedure than the previous one, allowing for a global analysis of all participants using metrics common across phases. In Phase 1, a sample size of 3 is used for initial technical validation to ensure the system functions as intended. This small sample size is sufficient for verifying the feasibility of the control system, ensuring that successful task completion is not due to random chance. Phase 2 involves five participants, enabling validation of the procedure and further technical validation of the system. Since statistical comparison of outcomes is not required at this stage, five participants provide a good balance between assessment thoroughness and time efficiency. The chosen sample sizes were determined based on best practices in literature (Kübler et al., 2014; Dillen et al., 2022; Xu et al., 2022).

Determining the optimal sample size for Phase 3 is challenging without prior knowledge of the standard deviations of quantitative measures. However, we can estimate it based on established usability engineering guidelines (Nielsen, 1994; Sauro and Lewis, 2016). Consequently, a sample size of 20 in Phase 3 is chosen to balance medium risk and fair precision, resulting in a 20% margin of error at a 90% confidence level. For metrics common to both Phases 2 and 3, combining data from both phases results in a total sample size of 25.

### 2.3 Experimental procedure

#### 2.3.1 Hardware and software

The hardware and software requirements for this study were previously determined during the design phase of the protocol. The following hardware recommendations are provided as guidelines, and any equipment with equivalent capabilities may be utilized. The software described has been previously implemented and tested. It may be refined if new requirements are identified during the initial piloting stages (Phases 1 and 2) of the study.

The control system under investigation combines AR and eye tracking with MI BCI. Using spatial awareness and object detection, the developed control system can propose context-aware actions to the user when they look at an object to provide a shared control strategy that enables complex actions while minimizing the number of MI classes that need to be decoded. Possible actions are displayed as holographic menus floating around the object, indicating which movement the participant should imagine to trigger the action. AR is also used to show virtual objects and display a simulated robot arm that is overlayed on top of real-world environments to enable a simplification of the required software components and faster iteration of prototypes. Two variants of the control system are available. The BCI variant uses eye tracking for selecting an object to interact with and BCI to select an action based on the currently selected object. Alternatively, the eye tracking variant uses eye tracking to select the action to perform in addition to the object to interact with.

Hence, an AR head-mounted display that supports these features is required. For this study, HoloLens 2 is used as holographic overlays are less intrusive, and the software development tools for HoloLens are the most mature among existing AR displays. The Unity 3D development platform (https://unity.com/) is used to develop the AR environment necessary for the experiments. Unity was chosen for its intuitive interface and extensive toolset for AR development. To facilitate the integration of AR-related components the Microsoft Mixed Reality Toolkit (https://learn.microsoft.com/en-us/windows/mixed-reality/mrtk-unity/mrtk2/?view=mrtkunity-2022-05) is used.

**Figure 2:**
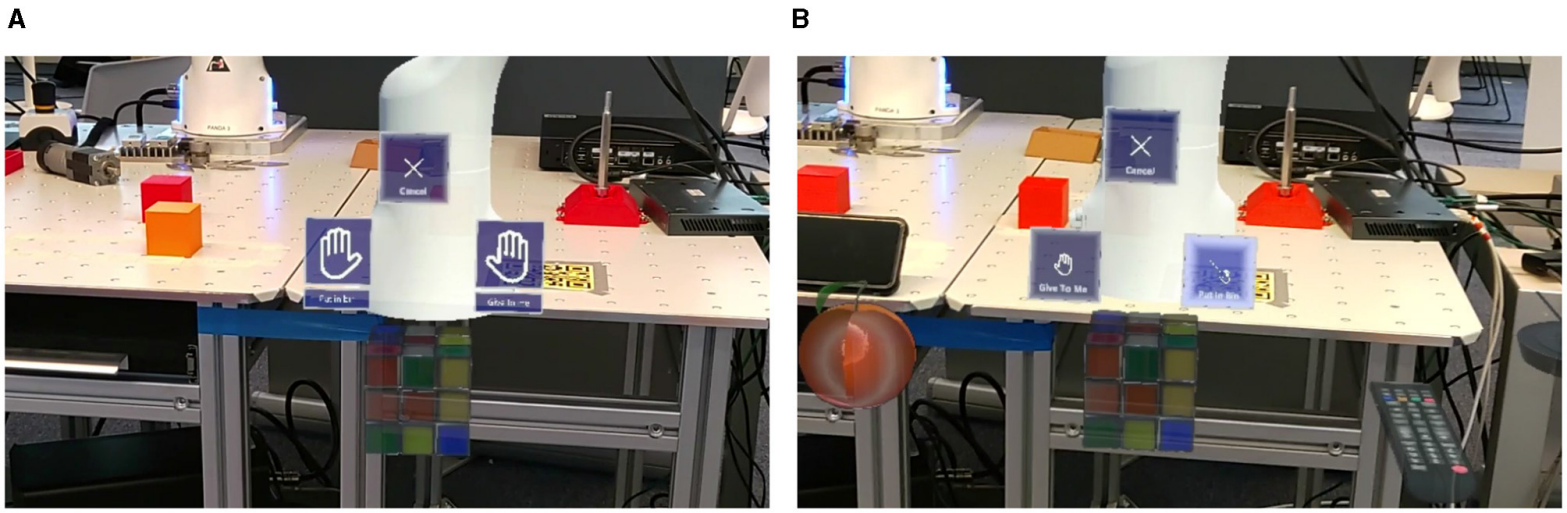
Example of the UI for when using the **(A)** BCI or **(B)** eye tracking control system variant.

The implemented environment simulates the robot and handles the logic related to the evaluation tasks. It also handles displaying the UI on top of the real world. Using a simulated environment facilitates keeping track of the state and position of the robot and interactive objects. Figure 2 shows examples of the UI during an evaluation task for the BCI and eye tracking control system variants, respectively.

Two devices are used for EEG acquisition in this study. To simulate commercial application scenarios and provide affordable options for individuals, we consider using an EEG device like the OpenBCI system (https://openbci.com/; OpenBCI, USA) with a passive gel-based EEG cap. The OpenBCI system offers a sampling rate of 250 Hz for 16 EEG channels and features open-source hardware and software, facilitating the integration of custom components with the control system. This device is utilized in Phase 1 to demonstrate the feasibility of using a consumer-grade device for BCI control, which is more representative of the type of device that would be used in real-world conditions.

To ensure high-quality EEG data suitable for further analysis, the Smarting ProX device from mBrainTrain (mbt, Serbia) is used. This device employs active wet electrodes and provides 64 channels with a sampling rate of up to 4,000 Hz. The Smarting ProX device is chosen for Phases 2 and 3 to ensure that the recorded EEG data can be thoroughly analyzed in subsequent investigations, focusing on MI patterns, fatigue-related markers, and visual processing evoked potentials.

The robot arm that is used in this protocol should be a collaborative robot that has built-in security measures to avoid potentially dangerous actions when a human is nearby. For this study, the Research series of robotic arms developed by Franka Robotics (https://www.franka.de/) is chosen. This robot is preferred due to its good safety rating, robust performance, and high versatility. The robot is operated with the robot operating system (ROS; https://www.ros.org/) by using pre-programmed scripts related to the evaluation task. Robot simulation is handled by a custom environment in Unity using the official URDF model published by the Emika company (https://github.com/frankaemika/). This allows for a direct translation of simulation to real-world robot control as the controller interface is the same for both the simulated and real robots.

To implement the software that handles training, testing, and loading the necessary ML models and other decoding pipeline components, the Python programming language (Van Rossum and Drake, 2009) is used. Python is preferred due to its free and open-source availability and widespread use in both ML and robotics programming. It has a wide variety of software libraries that facilitate the development of software that is necessary for this study. EEG processing is handled by the MNE library (Gramfort et al., 2013) and ML components are based on the Scikit-learn library (Pedregosa et al., 2011). Both libraries are chosen for their status as defacto options for their respective purposes. The real-time decoding and communication with other components is also implemented in Python using ZeroMQ (Akgul, 2013) for sharing messages between the system components. ZeroMQ is ideally suited for this purpose because it is lightweight, does not require external software, and provides a wide variety of clients in different programming languages, such as C#, the programming language that is used for scripting in Unity.

#### 2.3.2 Questionnaires

At the beginning of each session, participants complete questionnaires aimed at capturing potential confounding factors. During their initial visit, they fill out a questionnaire specifically designed to evaluate physiological factors that could influence MI activity. This questionnaire seeks information on the participant's age, sex, weight (in kilograms), height (in meters), and handedness.

To assess the aptitude of a participant at generating MI, the Motor Imagery Questionnaire version 3 (Malouin et al., 2007, MIQ-3) is used. For this questionnaire, participants perform a predefined set of MI tasks and rate how difficult they perceive performing MI to be for the given task. Participants complete this questionnaire at the beginning of their first session in all phases. Upon completing this questionnaire, the participant is also asked whether they prefer kinesthetic, internal visual, or external visual MI. Subjective mental fatigue is assessed with a visual analog scale (M-VAS) and physical fatigue is also assessed with a VAS (P-VAS). The participant is asked to indicate their fatigue levels on the scale at the beginning of each session and the end of the session to assess how much fatigue was induced using our control system. The indicated measure is then converted to a discrete scale from 0 to 100, enabling the quantitative comparison of the perceived level of fatigue induced by using our control system. Subjective fatigue was chosen as an outcome measure due to its potential impact on user experience.

During Phase 3, participants also indicate their fatigue levels between tasks involving the different system variants. Starting from Phase 2, the participants also fill in the Profile of Mood state (McNair et al., 1971, POMS) questionnaire at the beginning of each session to evaluate their current mood state. The POMS questionnaire consists of a list of 32 mood states where the participant has to indicate whether they currently experience the mood. The scale for the questionnaire for each mood state ranges from 0 to 4. This questionnaire was selected for its ability to quickly assess the current mood of the participant, which could be a confounding factor for MI decoding performance and thus influence the user experience.

At the end of each session, user experience is evaluated using the user experience questionnaire plus (Meiners et al., 2023, UEQ+), which is a modular extension of the well-established UEQ questionnaire (Laugwitz et al., 2008). This questionnaire provides quantitative outcomes for the subjective user experience component of usability, complementing the objective metrics that are presented in Section 2.4. Additionally, a semi-structured interview allows participants to give additional feedback. The questions that are used in this interview can be found as Supplementary material to this article. To ensure accurate transcripts of the user's responses, this interview is recorded with an audio recording device. If participant responses reveal major flaws in the design of the BCI control system or identify additional requirements, this feedback can be used to improve the control system between phases.

To enable comparison of the control system variants with and without BCI control, participants fill in the UEQ+ questionnaire for both variants separately. For more in-depth insights into the perceived differences in user experience between system variants, the interview will also include additional questions explicitly relating to the differences between the systems and the participant's preferences. Participants will also get the opportunity to suggest improvements at this stage.

#### 2.3.3 Tasks

At the start of each session, EEG data of users imagining the movements are acquired to train the ML models that decode user intention. Henceforth, these data will be denoted as calibration data. Three types of tasks are requested from the user to gather calibration data.

**Figure 3:**
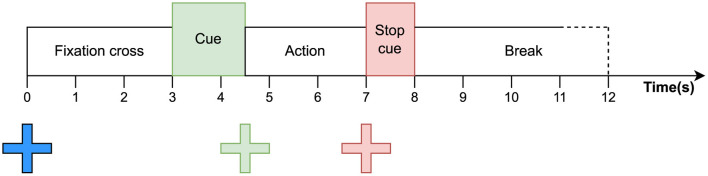
Timing of events for a single trial of a baseline EEG acquisition run.

**Baseline:** During a baseline run, the participant performs naturally occurring movements such as turning their head, blinking their eyes, and looking in different directions. The requested actions also include imagining the movements that will be used in the subsequent tasks. Possible movements include flexing the left hand or the right hand, pushing the tongue against the front teeth, and curling the toes of both feet. A randomized sequence consisting of each action repeated multiple times is requested. Each trial of a baseline run follows the procedure shown in Figure 3.

A run consists of five repetitions (called trials) of each baseline action. Each trial within a run starts by showing the participant a fixation cross, which they must fixate with their gaze while avoiding blinking and head- or eye movement. After 3 s, a textual cue appears at the center of the fixations cross instructing the participant which action they should perform. After 1.5 s, the fixation cross turns green, indicating that the participant should perform and maintain the requested action. A stop cue is given after 2.5 s by having the fixation cross turn red, indicating that the participant can stop their action. Finally, a break of randomized duration between 3 and 4 s precedes the next trial.

**Figure 4:**
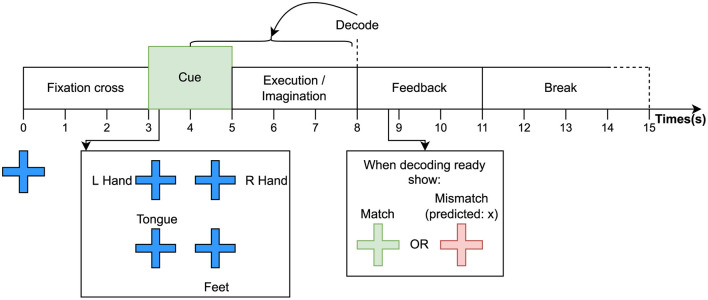
Timing of events for a single trial of an EEG acquisition run with textual feedback.

**Movement execution:** For this task, the participant is asked to execute movements while their signals are being decoded. The requested movement is communicated by an audiovisual cue. One run of this task consists of several cues with breaks in between, which are presented in randomized order. After the stop cue, feedback is given on whether the decoded movement matches the requested one. Feedback is communicated audiovisually in the form of text stating “match” or “mismatch” and a sound cue that indicates if the decoded movement matches the cue. At the end of each run, a score is shown with text stating the percentage of successfully decoded movements. Feedback is provided by an EEG decoding model that is trained with EEG data from the baseline run without feedback. Figure 4 shows the procedure for a single trial within a run and an example of textual feedback.

Identically to the baseline acquisition, the trial starts with a fixation cross. After 2 s, a cue is shown next to the cross for 2 s. When the fixation cross disappears, the participant holds the movement for 3 s. When the stop cue is displayed, the preceding 4 s of EEG data are sent to the decoding pipeline. A duration of 4 s was chosen for the decoding window to include motor preparation that occurs during the 1 s before the start cue is given. When the decoding result is ready, the participant receives feedback on whether the decoded movement matches the requested one. This step does not have a fixed time as we do not know exactly how long it takes to decode the movement. This depends on the hardware that is used for decoding and if any other software is running during the run. In the implementation that is used for our experiments, an upper limit of 3 s is set for decoding, as this is twice the longest decoding time encountered during the development of our decoding pipeline. Finally, a break of randomized duration between 3 and 4 s is given, followed by the next trial.

**Motor imagery:** For the MI task, the participant must imagine the requested movement instead of performing it. They also receive feedback on the correspondence between the decoded movement and the requested one, similar to the feedback provided for the executed movement task. The timings and procedure are the same as those used for movement execution runs (see Figure 4).

After completing the calibration tasks, the participant uses the system to complete tasks that are intended to assess the user experience and effectiveness of the proposed control system. To assess the added value of BCI, participants use one of the two variants of the control system to complete these tasks. The following tasks are performed to illustrate activities of daily living (Edemekong et al., 2024). The first two tasks are selected for their relevance in daily activities while the third task serves to motivate the participant through gamification.

**Object sorting:** This task is a simplified representation of daily tasks where objects need to be sorted, such as sorting laundry or garbage. The task was chosen due to its simplicity while still reflecting activities that paralyzed persons can not perform without assistance (Wilson et al., 2018). The participant is seated in front of a table with a robot arm attached to it and different colored baskets to their left, right, and front. At a fixed position in front of them, a cube of a specific color appears. Before the run, the participant is instructed to put the cube in the basket that matches the color of the cube. This is repeated 20 times with the cube color being randomized for each repetition.

**Drinking from a glass:** Drinking is one of the more challenging daily activities that require close collaboration between the human and the assistive robot (Edemekong et al., 2024). It mandates precise control of the robot and adds the challenge of avoiding spilling the liquid from the glass. For this task, the user sits at a table with a robot arm, several bottles containing different liquids, and several empty glasses with different shapes in front of them. They are instructed to complete several different scenarios that require them to fill specific glasses with specific liquids by following a fixed set of steps. For example, they need to use the control system to fill one of the glasses with orange juice and tell the robot to bring it to them so they can drink from it until it is empty and then tell the robot to put it in the dishwasher. The participant must complete a scenario 20 times, with the choice of the scenario being randomized at each repetition.

**Playing a board game:** Games allow paralyzed persons to entertain themselves while keeping their minds active. It is also more pleasurable for participants of the experiment and adds an element of competitiveness that could motivate participants to try their best at using the control system (Rapp et al., 2019). In this scenario, users are seated in front of a table and play a board game, such as chess for example. Starting from an initial game state, they can choose their moves and must reach a predefined end state. At the end of the session, they will also receive the opportunity to play a full game against an AI opponent.

#### 2.3.4 Session overview

**Figure 5:**
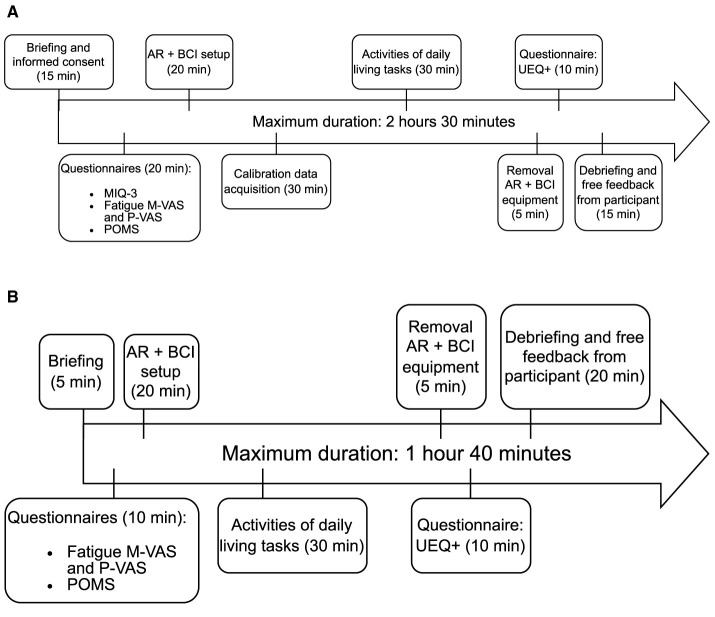
Phase 1 timelines for **(A)** session 1 and **(B)** session 2.

The procedure for Phase 1 consists of a validation experiment that follows the timeline presented in Figure 5. Timing estimates are provided for each step. Breaks are not shown on the timeline but are implicitly included in each task. Note that questionnaires at the beginning of the sessions and the equipment setup can be completed in parallel.

**Figure 6:**
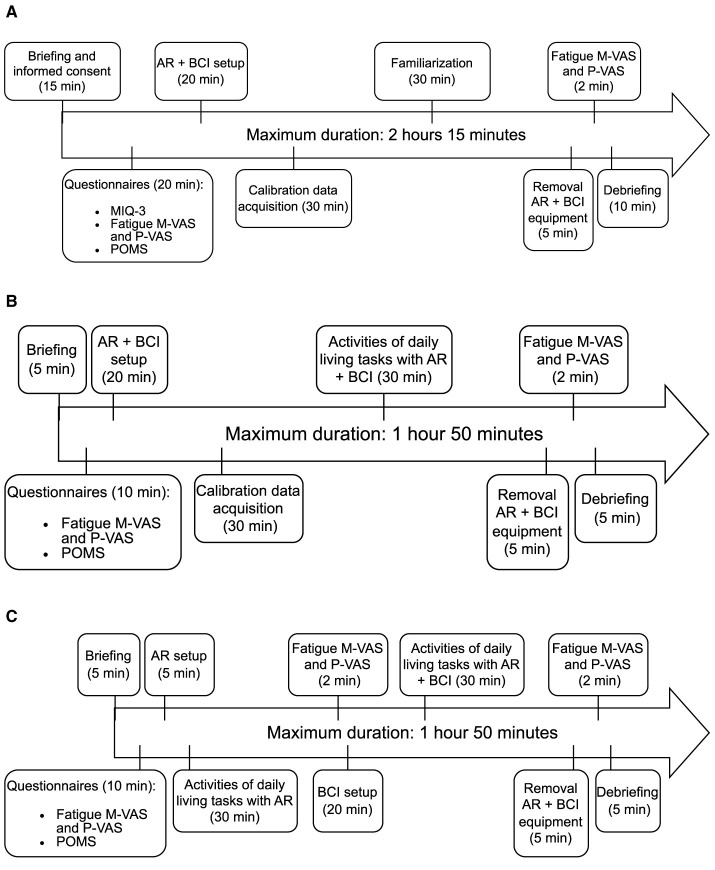
Phase 2 and 3 timelines for **(A)** session 1, **(B)** session 2, and **(C)** session 3.

Phases 2 and 3 consist of a full-scale user study that is more comprehensive than in Phase 1. The goal of this user study is to assess the user experience of the final control system prototype. The glass drinking and game tasks are introduced in addition to object sorting and participants now have to attend three sessions. Participants also use the alternative control system that uses eye-tracking only. The session structure is the same for both phases. Note that the order of the activities of daily living tasks with and without BCI in session 3 is interchangeable to avoid biasing the participant's judgment based on their previous experience with the other control system variant. The timings are shown in Figure 6.

### 2.4 Outcomes

The primary outcomes of this study are the objective system performance and subjective user experience. The metrics provided are designed to facilitate quantitative comparisons between BCI system prototypes and are commonly employed in user studies (Ortega-Gijon and Mezura-Godoy, 2019; Dillen et al., 2022) and ML benchmarks (Powers, 2008). Qualitative outcomes are intended to complement these metrics and guide future developments by identifying additional requirements.

Objective measures for the effectiveness of the system include the error rate when classifying movements (or inversely classifier accuracy) and the success rate of completing a task. Some tasks require the participant to complete the task within a limited amount of time and consider the task failed when this time runs out. For these tasks, the success rate is computed as


$$\begin{array}{l} success-rate=\frac{n_{c}}{n_{r}} \end{array}$$

where *n*_*c*_ is the number of completed runs and *n*_*r*_ is the total number of runs performed. The classifier accuracy is represented by measures such as balanced decoding accuracy, Cohen's Kappa, and F1 score. The formula used for computing balanced accuracy is


$$\begin{array}{l} balanced-accuracy=\frac{1}{2}\left(\frac{TP}{TP+FN}+\frac{TN}{TN+FP}\right) \end{array}$$

where *TP* is the number of true positives, *FN* is the number of false negatives, *TN* is the number of true negatives and *FP* is the number of false positives. Cohen's Kappa measures the agreement between two raters where one is a random classifier, i.e., 0.50 for a binary classifier, and our decoding pipeline (McHugh, 2012). The metric can be computed with the following formula


$$\begin{array}{l} \kappa =\frac{2\times \left(TP\times TN-FN\times FP\right)}{\left(TP+FP\right)\times \left(FP+TN\right)+\left(TP+FN\right)\times \left(FN+TN\right)} \end{array}$$

where *TP* is the number of true positives, *FN* is the number of false negatives, *TN* is the number of true negatives and *FP* is the number of false positives. The F1 score is the harmonic mean of the precision and recall (Powers, 2008) computed as


$$\begin{array}{l} F1=\frac{2\times TP}{2\times TP+FP+FN} \end{array}$$

where *TP* is the number of true positives, *FN* is the number of false negatives, and *FP* is the number of false positives. To measure efficiency, the task completion time and information transfer rate are used. The information transfer rate represents how fast users can achieve a specific goal based on the number of MI classes, the decoding time, and decoding accuracy. The definition and formula for this measure are presented in Sadeghi and Maleki (2019).

The main subjective measure of user experience is the UEQ+ questionnaire score. By aggregating these responses average scores for each aspect that is evaluated by the UEQ+ questionnaire can be obtained. Using the UEQ+ results from both control system variants enables the comparison of the overall difference in user experience by looking at the aggregated score. To investigate differences in specific areas of user experience, such as efficiency or dependability, specific scores of questionnaire items related to these aspects can be compared between the eye tracking and BCI system variants. Additionally, the responses from participants for the semi-structured interview provide further qualitative insights into the participant's perception of the control system.

The secondary outcome measures of this study relate to confounding factors that might influence the usage of the proposed control system. The outcome of the MIQ-3 questionnaire provides insights into the aptitude for MI of the participant by giving a score for MI proficiency that is calculated from the perceived difficulty values for each task. The POMS questionnaire outcomes will allow us to investigate whether the user's mood state will influence system performance and overall user experience by identifying correlations between mood states and BCI system performance. Additionally, physiological factors could show a large influence on the system performance or user experience outcomes. A strong influence of these factors suggests that further investigating the effects of strongly confounding factors could be worthwhile.

Finally, the recorded EEG data can be used for further investigation of the effects of using our control system on neural activity. Changes in EEG signals over sessions such as increased signal amplitude or stronger event-related synchronization or desynchronization could indicate training effects for MI aptitude. Alternatively, changes in EEG data during system usage can be associated with fatigue or a decrease in focus.

### 2.5 Data management

The EEG data are recorded as lab streaming layer (LSL Contributors, 2023) data streams using the LabRecorder application. The data are stored as Extensible Data Format files and organized using the Brain Imaging Data Structure format for the folder structure and file names (Gorgolewski et al., 2016). The recordings are stored on an access-controlled network attached storage which is only accessible to members of the research group. Only researchers who are directly involved in the project can view the dataset.

Questionnaire responses and outcome data are collected and managed using REDCap electronic data capture tools hosted at UZ Brussel (Harris et al., 2009, 2019). REDCap (Research Electronic Data Capture) is a secure, web-based software platform designed to support data capture for research studies, providing (1) an intuitive interface for validated data capture; (2) audit trails for tracking data manipulation and export procedures; (3) automated export procedures for seamless data downloads to common statistical packages; and (4) procedures for data integration and interoperability with external sources.

Personal information is processed according to the General Data Protection Regulation of the European Union. Any information linking the study outcomes to an individual is stored in an encrypted format and only accessible to researchers who are approved by the ethical committee. The data retention period for this dataset is 25 years. The anonymized data are available upon reasonable request.

### 2.6 Data analysis

To guarantee the adequacy of the obtained EEG data, an examination of visually evoked potentials (VEPs) is conducted when various cues are presented. The selection of VEP is based on its well-known characteristics and distinct patterns in the EEG signal (Creel, 2019). If the VEP is clearly discernible in the occipital sensors (EEG channels O1 and O2), the signal is deemed to be of satisfactory quality. Additionally, the data quality assessment features of the PyPREP pipeline (Appelhoff et al., 2022) are used to ensure that the other EEG sensors are also of sufficient quality.

Subsequently, the EEG data are used to train ML pipelines for MI decoding. Based on markers that are recorded during the experiment, windows of EEG data are extracted that correspond to an MI class of interest. After acquiring 15 examples for each imagined movement, the ML pipeline is trained on the resulting dataset. The number of samples per class was determined in previous research as the minimal amount required for training an ML pipeline while minimizing the time that is taken for the acquisition of calibration data (Dillen et al., 2023). These pipelines are used to classify imagined movements from EEG signals in real-time. Candidate pipelines include using Common Spatial Patterns (Congedo et al., 2016) for feature extraction and linear discriminant analysis (McLachlan, 1992) for classification or deep learning approaches. The deep learning approaches under consideration are the Shallow ConvNet that was created by Schirrmeister et al. (2017) and the EEGNet model from Lawhern et al. (2016).

The average classification accuracy of the MI decoding models is compared with a Wilcoxon signed rank test after performing 100 different cross-validation splits. This ensures that any differences in performance between decoding pipelines with different settings are statistically significant and that results are not due to random chance in the way the dataset was split into training and test data. The comparison between control system variants uses the quantitative outcome measures from questionnaires and system performance metrics. Here, for each measure under analysis, a Shapiro-Wilk test is first used to verify if the data is normally distributed. If the data are normally distributed, a paired samples *t*-test is used to investigate if average outcomes are significantly different between control system variants. When the data are not normally distributed, the test of choice is the Wilcoxon signed-rank test.

The assessment of the influence of confounding factors is achieved by first splitting the outcome data into groups based on the value of the factor under investigation. The normality of the data distribution is first verified with a Shapiro-Wilk test. For normally distributed data, an independent samples *t*-test is used to compare the outcomes between groups. When the data are not normally distributed, the Wilcoxon rank-sum test is used. If the differences between groups based on a chosen factor are statistically significant, further investigation of the impact of this factor in future work is warranted.

## 3 Discussion

The goal of this study protocol is to evaluate the real-world usability of a non-invasive MI-based BCI control system prototype utilizing AR and eye tracking. Additionally, the protocol aims to assess the added value of using BCI as a control method for assistive robots, in comparison to a control system based solely on eye tracking. Most BCI research limits itself to the offline evaluation of decoding pipelines or simply validates the technical feasibility of the developed application (Dillen et al., 2022). However, a user-centered approach that follows best practices of human-computer interaction research, is necessary to ensure that the BCI application is usable in real-world use cases (Dix, 2008; Garro and McKinney, 2020).

The majority of non-invasive BCI control systems employ a graphical UI that is displayed on a computer screen (Yang et al., 2018; Kuhner et al., 2019; Jeong et al., 2020). However, previous research has shown that the inclusion of AR in a BCI control system can result in a system that is more mobile and allows the superposition of the UI on top of the real environment while providing real-time feedback to the user (Si-Mohammed et al., 2020; Sanna et al., 2022). Furthermore, combining eye tracking with BCI provides an intuitive multi-modal UI (Xu et al., 2022). Thus, our control system provides a shared control AR UI that integrates BCI and eye tracking as interaction modalities and uses spatial mapping and object detection to propose environment-aware actions.

The user study protocol presented in this publication evaluates the usability of the system by assessing the user experience of our BCI control system and comparing the system performance with alternative control strategies that do not use BCI. This allows for the assessment of the added value of combining AR with BCI and eye tracking in a shared control system. Building upon well-established guidelines for quantitative user studies (Sauro and Lewis, 2016), the protocol takes an iterative approach that expands the user study in each consecutive phase and facilitates improvements to the design of the control system between each phase. The protocol is intended to be versatile and enable the evaluation of a wide variety of BCI applications that involve the control of an electronic device. Using this protocol should allow researchers to focus on the technical aspects of BCI control systems under development.

One notable limitation of this study is its inadequacy to confirm whether the usability outcomes that are observed in able-bodied individuals extend to other target populations. Additionally, the study, although representative of daily activities, falls short of ensuring direct applicability to real-world scenarios. To address these limitations in future research, it is crucial to conduct a user evaluation specifically tailored to other target populations, employing the same protocol. Moreover, conducting follow-up in-field user studies will be essential to validate the control system more comprehensively by providing users with extended, long-term access.

The current experimental procedure can also easily be modified or expanded. Including more alternative control systems, such as steady-state visually evoked potential BCI or electromyography-based control for example would enable an extensive benchmark of different alternatives to identify the most suited approach for a specific use case. Introducing additional tasks such as assembly or 3D environment navigation could strengthen the significance of findings on the differences in performance outcomes and ensure that the system can optimally assist users in their daily lives. Additionally, the same procedure could be used to target BCI applications that are intended for healthy individuals such as control of a virtual avatar or human-robot collaboration in industrial settings.

Another future perspective includes a detailed investigation of confounding factors. This opens up the possibility of designing system variants that account for differences between individuals or designing custom systems for different population groups depending on these factors. For example, if low MI aptitude is found to hinder usability, the system could switch to a different EEG paradigm such as steady-state visually evoked potential, which relies on induced neural responses rather than the user's focused mental activity, for participants with low MI aptitude. Another possibility could be to automatically select the optimal decoding model based on specific user attributes if it is found that this improves real-time MI decoding accuracy.

## 4 Ethics and dissemination

The protocol is approved by the Medical Ethics Committee of UZ Brussel and VUB (BUN1432023000232) and adheres to the principles of the Declaration of Helsinki for medical research involving human participants (World Medical Association, 2013). There are no additional ethical considerations for this study. Participants provide their written informed consent before starting their first session.

The findings of this study will be disseminated through journal articles focused on the specific outcome measures. Two articles are currently planned: one will address the objective performance of effectiveness and efficiency to validate the BCI control system, while the other will discuss user experience outcomes and compare results between the eye-tracking and BCI system variants. Additionally, VUB and CY universities will issue a press release to promote the research to a wider audience. Finally, demonstration videos and pictures will be produced for further dissemination on social media and university news channels.

## Ethics statement

The studies involving humans were approved by the Medical Ethics Committee of UZ Brussel and VUB. The studies are conducted in accordance with the local legislation and institutional requirements. The participants provide written informed consent to participate in this study.

## Author contributions

AD: Conceptualization, Data curation, Formal analysis, Investigation, Methodology, Software, Validation, Visualization, Writing – original draft, Writing – review & editing. MO: Conceptualization, Methodology, Software, Validation, Visualization, Writing – original draft, Writing – review & editing. MD: Conceptualization, Formal analysis, Methodology, Validation, Writing – review & editing. FG: Conceptualization, Funding acquisition, Project administration, Supervision, Validation, Writing – review & editing. BR: Project administration, Resources, Supervision, Validation, Writing – review & editing. BV: Methodology, Supervision, Validation, Writing – review & editing. OR: Conceptualization, Funding acquisition, Methodology, Resources, Supervision, Validation, Writing – review & editing. KD: Conceptualization, Funding acquisition, Investigation, Methodology, Project administration, Resources, Supervision, Validation, Writing – original draft, Writing – review & editing.
