# Supplementary material for: Evaluating the real-world usability of BCI control systems with augmented reality: a user study protocol
Source: Front Hum Neurosci. 2024 Aug 5;18:1448584. doi: 10.3389/fnhum.2024.1448584 (PMC11330773; doi:10.3389/fnhum.2024.1448584)
Supplement: Supplementary file 1 [file Data_Sheet_1.PDF]

Questions for the user interview

Did you feel like you were able to successfully complete the tasks with the BCI control system?

Did you feel like you were in control of the robotic arm?

Do you think that BCI provides an added value to the control system, assuming that you are not able to move or talk?

Which control system variant did you prefer? (Only when eye tracking and BCI were compared)

Do you think a different approach would work better? If yes, what do you suggest?

Do you have any suggestions for improving the current control system?
